# Polymorphisms in the Haem Oxygenase-1 promoter are not associated with severity of *Plasmodium falciparum* malaria in Ghanaian children

**DOI:** 10.1186/s12936-015-0668-5

**Published:** 2015-04-11

**Authors:** Helle H Hansson, Lasse Maretty, Christina Balle, Bamenla Q Goka, Elisa Luzon, Francis N Nkrumah, Mette L Schousboe, Onike P Rodrigues, Ib Christian Bygbjerg, Jørgen AL Kurtzhals, Michael Alifrangis, Casper Hempel

**Affiliations:** Centre for Medical Parasitology at Department of Immunology & Microbiology, University of Copenhagen, Østerfarimagsgade 5, Building 22-23, 1014 Copenhagen K., Denmark; Department of Clinical Microbiology and Department of Infectious Diseases, Copenhagen University Hospital (Rigshospitalet), Copenhagen, Denmark; Department of Child Health, Korle Bu Teaching Hospital, PO Box KB 77, Korle Bu, Accra, Ghana; Noguchi Memorial Institute for Medical Research, PO Box LG 581, Legon, Ghana

**Keywords:** HMOX1, Falciparum malaria, Polymorphisms, Severe malaria

## Abstract

**Background:**

Haem oxygenase-1 (HO-1) catabolizes haem and has both cytotoxic and cytoprotective effects. Polymorphisms in the promoter of the *Haem oxygenase-1* (*HMOX1*) gene encoding HO-1 have been associated with several diseases including severe malaria. The objective of this study was to determine the allele and genotype frequencies of two single nucleotide polymorphisms; A(−413)T and G(−1135)A, and a (GT)_n_ repeat length polymorphism in the *HMOX1* promoter in paediatric malaria patients and controls to determine possible associations with malaria disease severity.

**Methods:**

Study participants were Ghanaian children (n=296) admitted to the emergency room at the Department of Child Health, Korle-Bu Teaching Hospital, Accra, Ghana during the malaria season from June to August in 1995, 1996 and 1997, classified as having uncomplicated malaria (n=101) or severe malaria (n=195; defined as severe anaemia (n=63) or cerebral malaria (n=132)). Furthermore, 287 individuals without a detectable *Plasmodium* infection or asymptomatic carriers of the parasite were enrolled as controls. Blood samples from participants were extracted for DNA and allele and genotype frequencies were determined with allele-specific PCR, restriction fragment length analysis and microsatellite analysis.

**Results:**

The number of (GT)_n_ repeats in the study participants varied between 21 and 46 with the majority of alleles having lengths of 26 (8.1%), 29/30 (13.2/17.9%) and 39/40 (8.0/13.8%) repeats, and was categorized into short, medium and long repeats. The (−413)T allele was very common (69.8%), while the (−1135)A allele was present in only 17.4% of the Ghanaian population. The G(−1135)A locus was excluded from further analysis after failing the Hardy-Weinberg equilibrium test. No significant differences in allele or genotype distribution of the A(−413)T and (GT)_n_ repeat polymorphisms were found between the controls and the malaria patients, or between the disease groups, for any of the analysed polymorphisms and no associations with malaria severity were found.

**Conclusion:**

These results contribute to the understanding of the role of *HMOX1*/HO-1. This current study did not find any evidence of association between *HMOX1* promoter polymorphisms and malaria susceptibility or severe malaria and hence contradicts previous findings. Further studies are needed to fully elucidate the relationship between *HMOX1* polymorphisms and malarial disease.

## Background

In malaria patients, a large number of infected erythrocytes rupture in the bloodstream, releasing considerable amounts of erythrocyte haemoglobin [1], which is oxidized and releases its haem moiety [2]. This results in large quantities of free haem, which can be highly cytotoxic to both host cells and parasites [1-3]. Survival of the host relies in part on ability to prevent the cytotoxic and inflammatory effects of the free haem. Free haem is only found under pathological conditions because excess haem is usually removed via the microsomal haem degradation (MHD) pathway. However, this pathway may become saturated in situations with large amounts of free haem [3,4]. The rate-limiting enzyme of the MHD pathway is Haem oxygenase (HO) [5]. Two isoforms (HO-1 and HO-2) have been characterized and are expressed in humans. HO-1 is the inducible isoform, whereas HO-2 is the constitutive isoform [6]. HO-1 is a highly inducible 32 kDa protein, with the highest activity in spleen, liver and bone marrow, where senescent erythrocytes are sequestered and degraded [7,8]. The HO-1-encoding gene *HMOX1* is located on chromosome 22q12 [9], is approximately 14 kb long, and organized into 4 introns and 5 exons [7]. Transcriptional control of *HMOX1* is governed by multiple regulatory elements localized in the promoter of the gene, as well as by enhancers, responsible for *HMOX1* induction in response to increased haem concentration [10,11]. HO-1 catabolizes free haem into biliverdin (that is immediately converted to bilirubin), releasing ferrous iron (that is sequestered into the iron storage protein ferritin) and carbon monoxide (CO) [8]. All of these have been associated with the cytoprotective effects of HO-1. Indeed, bilirubin is a potent and abundant antioxidant in mammalian tissue [12,13] and ferritin is a cytoprotective molecule [14,15], whereas CO may affect the regulation of apoptosis [16-18], and inflammation, and has been suggested to mimic the cytoprotective effects of HO-1 [19]. However, high levels of HO-1 related products might also have damaging effects, resulting in an overall pro-oxidant effect, being cytotoxic and causing tissue damage [5,15,20-22]. Although both protective and damaging properties of HO-1 have been shown, HO-1 is essential and HO-1 deficiency leads to severe illness and death in both humans and mice [23-25].

Humans have been shown to differ quantitatively in their *HMOX1*/HO-1 activity due to polymorphisms in the *HMOX1* promoter [26-29]. Two single-nucleotide polymorphisms (SNPs); T(−413)A and G(−1135)A, and a (GT)_n_ repeat length polymorphism in the *HMOX1* promoter have been described [7,27,28]. The T(−413)A SNP has been shown to influence promoter activity, with the A allele having a significantly higher activity *in vitro* compared to the T allele [27], while the functional importance of the G(−1135)A SNP is still unknown [28,30]. Finally, the (GT)_n_ repeat length polymorphism has been described in several studies in various ethnic populations with repeat size varying from 13 to 45 repeats and main alleles at 23, 30 and 39 repeats [27,31-35]. This purine-pyrimidine alternating sequence can result in a Z-DNA conformation and negatively affect transcriptional activity [36,37]. (GT)_n_ repeat length polymorphisms have been associated with many different diseases, including diabetes, cardiovascular, pulmonary, and neurological disease as reviewed by Exner *et al.*) and by Garcia-Santos & Chies, where long (GT)_n_ repeats, associated with lower HO-1 expression, were identified as risk factors [38,39].

Several studies have investigated a possible association between HO-1 and *Plasmodium falciparum* infections and have demonstrated both increased expression of HO-1 during malaria infection [40-42] and associations between *HMOX1* promoter polymorphisms and malaria disease severity [32-35,43,44]. In mice, an up-regulation of HO-1 was associated protection against cerebral malaria, whereas associations between the short, highly inductive (GT)_n_ repeat alleles and risk of severe malaria have been shown in human studies in both The Gambia, Myanmar, and Angola [32-34]. A lack of association between malaria severity and length of GT repeats has been documented in Thailand [35]. Still, the role of HO-1 during malaria remains unclear [44].

In the present study, the presence of the two single-nucleotide polymorphisms (T(−413)A and G(−1135)A) and the length of the (GT)_n_ repeat were assessed in 583 Ghanaian children with malaria from 0–15 years of age to search for possible associations with malaria disease susceptibility and severity.

## Methods

### Study population

The study population consisted of patients admitted to the Department of Child Health, Korle-Bu Teaching Hospital, Accra, Ghana during the malaria season (June to August) in 1995, 1996 and 1997, as described in detail in earlier publications [45,46]. All patients were children between 0 and 14 years of age who fulfilled the general inclusion criteria of an asexual *P. falciparum* parasitaemia of more than 10,000 parasites/μl, and an axillary temperature of more than 37.5°C. A total of 296 malaria patients were enrolled; 101 with uncomplicated malaria and 195 with severe malaria (defined as severe anaemia (n=63) or cerebral malaria (n=132)). Patients with a positive sickling test or any other disease than malaria were excluded as a criterion used in the study the samples were originally collected for. Blood samples from healthy sickle cell-negative children between 0 and 15 years of age were collected as control samples from Dodowa, a nearby community (n=287). Both patients and controls were included after signed consent from patients or guardians after receiving standardized information in local language. Both patient and control population is a mixture of several ethnic groups, possibly with a slightly more uniform population in Dodowa. However, Ga-Adangme is the dominating ethnic group in both populations. The study population is well described with very thorough patient characterization and has been studied extensively, among other things, with regards to mannose-binding lectin genotypes [45] and complement receptor 1 [47]. The study was approved by the ethics and protocol review committee at the University of Ghana Medical School and the Ministry of Health, Ghana.

### DNA extraction and whole genome amplification

Upon admission, venous blood samples were collected in EDTA-containing test tubes. Within two hours after collection, plasma was separated, and pellets frozen at −20°C. Genomic DNA was extracted as described previously [48]. Whole genome amplification of the extracted products was performed with Repli-g Mini Kits (Qiagen, Copenhagen, Denmark).

### Determining SNPs in the *HMOX1* promoter

Two SNPs were analysed in the *HMOX1* promoter; the T(−413)A and G(−1135)A. A simple allele-specific PCR was developed to detect the T(−413)A SNP. Two primer-pairs were designed to amplify a 307-bp target sequence based on the nucleotide sequence of Genbank S58267, sharing the same forward primer, and only differing in the 3’-nucleotide end of the reverse primers, making them allele specific. Furthermore, to increase the specificity of the allele-specific primers, a mismatch near the 3’ends were introduced (Table 1). The PCR products were subsequently analysed by 1.5% agarose gel electrophoresis.

**Table 1:** **Primer sequences and conditions for the polymerase chain reactions (PCRs)**

**Allele specific PCRs**	**Sequences**
Fw	5′-ACTGGCACTCTGCTTTATGTGTGA-3′
Rw A(−413)	5′-GGAGGCAGCGCTGCTCAGAG*T*AAT-3′
Rw (−413)T	5′-GGAGGCAGCGCTGCTCAGAG*T*AAA-3′
Conditions	**95°C 15 min, 35 cycles: (94°C 30 sec, 60°C 30 sec, 72°C 30 sec), 72° 10 min**
**Restriction fragment length polymorphism**	
Fw	5′-TTATTTTATATTTTGTAGAG*C*C-3′
Rw	5′-AGATGATTCATACAGTCCTTTC-3′
Conditions	**94°C 15 min, 45 cycles: (94°C 30 sec, 49°C 30 sec, 72°C 3 min), 72° 10 min**
**(GT)**_**n**_ **repeat length polymorphism**	
Fw **(5’fam)**	5′-AGAGCCTGCAGCTTCTCAGA-3′
Rw	5′-ACAAAGTCTGGCCATAGGAC-3′
Conditions	**95°C 15 min, 30 cycles: (95°C 30 sec, 64°C 30 sec, 72°C 30 sec), 72° 10 min**

Primer sequences and conditions for the PCR reactions used to determine the polymorphisms in the *HMOX1* promoter. The primer pairs for the allele specific PCRs and the restriction fragment length PCR contains a mismatch *(Italic)*. The forward primer for the (GT)_n_ repeat length PCR is fluorescein-conjugated (highlighted). Primers for the allele specific PCRs were designed based on the *HMOX1* nucleotide sequence (Genbank S58267). The primers for the restriction fragment length PCR and (GT)_n_ repeat length PCR were designed by He *et al.* [30] and Takeda *et al.* [33], respectively.

A restriction fragment length polymorphism (RFLP) analysis was used to detect the G(−1135)A SNP. Primers, as described elsewhere [30], contained a mismatch (see Table 1), which creates a restriction site in the amplified product if the G allele is present. The PCR products were then digested overnight with the restriction enzyme *HpaII* at 37°C and visualized on a 1.5% agarose gel, showing one band of 225 bp (homozygote for the A allele), two bands of 23 and 202 bp (homozygote for the G allele) or all three bands (heterozygote).

For both PCR protocols, one μl DNA extract was amplified in a 20 μl reaction mix consisting of 4.0 μl H_2_O, 5.0 μl 2.0 μM primermix and 10.0 μl TEMPase Hot Start (Ampliqon, Odense, Denmark). The reactions were performed in a 96-well PCR plate (Starlab GmbH, Hamburg, Germany) in a VWRi Duo Cycler (VWR/Bie&Berntsen, Radnor, PA, USA). Conditions for amplification are provided in Table 1. Selected samples were sequenced by Sanger to verify the genotyping of the two SNPs.

### Determining the *HMOX1* promoter (GT)_n_ repeat length polymorphism

A PCR product containing the (GT)_n_ repeat was amplified with a fluorescein-conjugated forward primer and an unlabelled reverse primer designed by Takeda *et al*. [33] One μl DNA was amplified in a 10 μl reaction consisting of 1.5 μl H_2_O, 2.5 μl 1.0 μM primermix and 5.0 μl TEMPase Hot Start DNA Polymerase (Ampliqon, Odense, Denmark). The reactions were performed in a 96-well PCR plate (Starlab GmbH, Hamburg, Germany) in a VWRi Duo Cycler (VWR/Bie&Berntsen, Radnor, PA, USA). Primer sequences and amplification conditions are provided in Table 1. One μl PCR product was added to a 10.5 μl reaction containing 9.25 μl HiDi formamide^TM^ (Applied Biosystems, Foster City, CA, USA) and 0.25 μl GeneScan^TM^ 500 LIZ^TM^ Dye Size Standard (Applied Biosystems, Foster City, CA, USA) in a 96 well MicroAmp® Optical Reaction Plate (Applied Biosystems, Foster City, CA, USA) and denatured for 3 minutes at 95°C before analysis with an ABI 3730 XL Genetic Analyzer (Applied Biosystems, Foster City, CA, USA). Subsequent allele scoring of the microsatellites was performed using GeneMapper version 4.1 (Applied Biosystems, Foster City, CA, USA). Alleles were divided into short repeats “S” (<27 repeats), medium “M” (27–32 repeats) and long “L” (>32 repeats) based on earlier classifications [26,34,49]. Selected samples were sequenced to verify the determination of repeats.

### Statistical analysis

Deviations from the Hardy-Weinberg equilibrium at the two loci; T(−413)A and G(−1135)A were tested using The Court Lab Calculator [50]. Cut-off was set to p < 0.05. Allele and genotype frequencies were compared between the disease groups with Chi-square or Fisher’s exact test (SigmaPlot 12.3 SPSS Inc., USA) for the SNPs and the repeat length polymorphism. Associations between alleles, genotypes, or haplotypes, and disease groups were investigated with logistic regression models to determine odds ratio and p values, with disease group as outcome variable (defined as controls, uncomplicated malaria patients, and severe malaria patients (collectively and divided into severe anaemia and cerebral malaria). Age and gender were included as covariates; p-values < 0.05 were considered significant. Calculations were performed using SAS ver. 9.2, (2002–2008, SAS Institute Inc., Cary, NC, USA). Linkage disequilibrium was calculated using Arlequin [51].

## Results

### Population demographics

In total, blood samples were collected from 287 controls and 296 patients; 101 with uncomplicated malaria, and 195 with severe malaria (defined as severe anaemia (n=63) or cerebral malaria (n=132)). All patients were children 0–15 years of age (see Table 2).

**Table 2:** **Demographics of the study population**

	**Controls (n=287)**	**Uncomplicated malaria (n=101)**	**Severe anaemia (n=63)**	**Cerebral malaria (n=132)**
**Age (years)**				
Mean ± SD	8.01 ± 3.97	5.51 ± 3.32	2.92 ± 2.62	4.81 ± 2.77
Minimum/Maximum	0-15	0.5-14	0-12	0.5-13
**Sex (n)**				
Male	145 (50.52%)	55 (54.46%)	40 (63.49%)	69 (52.27%)
Female	142 (49.48%)	46 (45.54%)	23 (36.51%)	63 (47.72%)
**Parasitaemia (p/ul)**				
Median	-	52.000	50.265	97.157
Percentiles 25 and 75	-	24.941-121.912	17.730-114.295	33.788-212.900
**Haemoglobin (g/dl)**				
Mean ± SD	-	10.49 ± 1.89	4.10 ± 1.17	7.56 ± 2.26
Minimum/Maximum	-	6.80-17.5	1.80-11.20	0.80-13.40

Demographic data for the Ghanaian control, uncomplicated malaria, severe anaemia and cerebral malaria groups.

### Allele and genotype frequencies of the SNPs in the *HMOX1* promoter

The allele-specific PCR and RFLP analysis successfully determined the allele distribution of the T(−413)A SNP in 556 of 583 samples (95.4%) and the G(−1135)A SNP in 478 of 583 samples (82.0%), respectively. Results were confirmed by sequencing of selected samples representing the six possible genotypes. As can be seen in Table 3, the T(−413) allele was common in the study population (69.8%), reflected in high frequencies of both the heterozygote A/T (41.0%) and homozygote T/T (49.3%) genotype. The G(−1135) allele was common (82.6%) with a frequency of 75.1% of the homozygote genotype G/G and 15.1% of the heterozygote G/A. The T(−413)A genotype distribution was found to be in Hardy-Weinberg equilibrium (Χ^2^=0.42, p=0.51), whereas, the G(−1135)A genotype distribution was not in equilibrium, (Χ^2^=107.9, p < 0.0001) and this SNP was therefore excluded from further analysis. Analysis of the T(−413)A alleles and genotype frequencies showed no significant differences between the control, uncomplicated or severe malaria (cerebral malaria, severe anaemia and total) groups (p > 0.3 in all cases). Furthermore, logistic regression, adjusted for age and sex, showed no significant association between the T(−413)A alleles or genotypes and severity of malaria (p > 0.4 in all cases).

**Table 3:** **Prevalence of the T(−413)A and G(−1135)A alleles and genotypes**

**T(−413)A Alleles and genotypes N (%)**		**Controls (n=281)**	**Uncomplicated malaria (n=95)**	**Severe anaemia (n=61)**	**Cerebral malaria (n=119)**
**Alleles**	**A**	175 (31.1)	56 (29.5)	35 (28.7)	70 (29.4)
	**T**	387 (68.9)	134 (70.5)	87 (71.3)	168 (70.6)
**Genotypes**	**A/A**	26 (9.3)	11 (11.6)	5 (8.2)	12 (10.1)
	**A/T**	123 (43.8)	34 (35.8)	25 (41.0)	46 (38.7)
	**T/T**	132 (47.0)	50 (52.6)	31 (50.8)	61 (51.3)
**G(−1135)A Alleles and genotypes N (%)**		**Controls (n=238)**	**Uncomplicated malaria (n=78)**	**Severe anaemia (n=55)**	**Cerebral malaria (n=107)**
**Alleles**	**G**	397 (83.4)	123 (78.9)	91 (82.7)	179 (83.6)
	**A**	79 (16.6)	33 (21.2)	19 (17.3)	35 (16.4)
**Genotypes**	**G/G**	179 (75.2)	57 (73.1)	42 (76.4)	81 (75.7)
	**G/A**	39 (16.4)	9 (11.5)	7 (12.7)	17 (15.9)
	**A/A**	20 (8.4)	12 (15.4)	6 (10.9)	9 (8.4)

Prevalence of alleles and genotypes in controls, uncomplicated malaria, severe anaemia and cerebral malaria groups. No significant differences in the allele or genotype distribution were found between any of the groups (p > 0.05. G(−1135)A was excluded from further analysis since the control group failed the Hardy-Weinberg equilibrium test.

### Allele and genotype frequencies of the (GT)_n_ repeat length polymorphism in the *HMOX1* promoter

**Figure 1:**
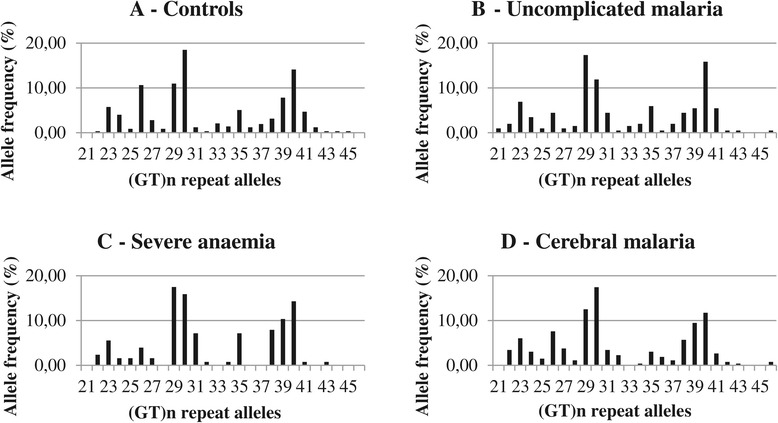
Frequency distribution of the (GT)_n_ repeats in the study groups. Frequency distribution of the (GT)_n_ repeat alleles in the four study groups.1**A**: The control group. 1**B**: The uncomplicated malaria group. 1**C**: The severe anaemia group. 1**D**: The cerebral malaria group.

The (GT)_n_ repeat length polymorphism were successfully genotyped in 572 of 583 samples (98.1%). Sequencing of selected samples was performed to define the size of the repeats (data not shown). The distributions in the control, uncomplicated malaria, severe anaemia and cerebral malaria groups are shown in Figure 1. In total, twenty-six (GT)_n_ alleles were identified, ranging from 21 to 46 repeats. The majority of alleles had lengths of 26 (8.1%), 29/30 (13.2/17.0%) or 39/40 (8.0/13.8%) GT repeats. The alleles were categorized into: short “S” (<27 repeats), medium “M” (27–32 repeats), or long “L” (>32 repeats). Based on these three categories, the patients were classified as having a S/S, S/M, S/L, M/M, M/L or L/L genotype. The allele and genotype frequencies are shown in Table 4. The long repeat alleles (L) were the most prevalent in all four study groups ranging from 42.6 to 45.7%, with high frequencies of the M/L (23.2-41.4%) and L/L (19.5-22.7%) genotypes, whereas the short repeat alleles (S) were found with the lowest frequencies, ranging from 19.1 to 21.6%, and genotype frequencies of 4.3-6.3% (S/S) and 12.6-18.0% (S/M). The distributions of alleles and genotypes were compared between the groups; controls, uncomplicated malaria patients, severe anaemia and cerebral malaria patients (and as a combined severe malaria patient group of severe anaemia and cerebral malaria). No significant differences were found between any of the groups (p > 0.5 in all cases). Logistic regression models were used to test for an association between the alleles or genotypes and severity of malaria. All models were adjusted for age and sex. No significant associations were found (p > 0.7 in all cases). Furthermore, the data was analysed comparing genotypes containing at least one “L” allele (S/L, M/L, L/L) against non-L carriers (S/S, S/M, M/M) and no significant differences were found (p > 0.9).

**Table 4:** **Prevalence of the categorized (GT)**_**n**_ **repeat alleles and genotypes**

**Microsatellites Alleles & genotypes N (%)**		**Controls (n=564)**	**Uncomplicated malaria (n=95)**	**Severe anaemia (n=58)**	**Cerebral malaria (n=128)**
**Alleles**	**S**	114 (20.2)	41 (21.6)	23 (19.8)	48 (18.8)
	**M**	210 (37.2)	68 (35.8)	40 (37.5)	100 (39.1)
	**L**	240 (42.6)	81 (42.6)	53 (45.7)	108 (42.2)
**Genotypes**	**S/S**	12 (4.3)	6 (6.3)	3 (5.2)	6 (4.7)
	**S/M**	41 (14.5)	12 (12.6)	6 (10.3)	23 (18.0)
	**S/L**	49 (17.4)	17 (17.9)	11 (19.0)	13 (10.2)
	**M/M**	44 (15.6)	17 (17.9)	5 (8.6)	20 (15.6)
	**M/L**	81 (28.7)	22 (23.2)	24 (41.4)	37 (28.9)
	**L/L**	55 (19.5)	21 (22.1)	9 (15.5)	29 (22.7)

Frequencies of the alleles and genotypes of the categorized (GT)_n_ repeat alleles in Short “S” (<27), Medium “M” (27–32) and Long “L” (>32). No significant differences in the allele or genotype distribution were found between any of the groups (p > 0.05).

### Analysis of the combination of the A(−413)T and (GT)n repeat length polymorphisms

**Figure 2:**
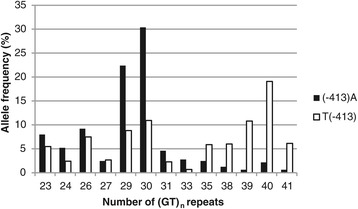
Allele frequencies of the T(−413)A single nucleotide polymorphism for each (GT)_n_ repeat length polymorphism. Frequency of the T(−413)A single nucleotide polymorphism for each (GT)_n_ repeat length. The A(−413) allele is shown in closed bars, the (−413)T in open bars. (GT)_n_ repeats with allele frequencies of less than 2% of both alleles of the A(−413)T single nucleotide polymorphism are not shown.

The T(−413)A and (GT)n repeat alleles were next considered together, and the frequencies are shown in Figure 2. (−413)A/(GT)_29_, (−413)A/(GT)_30_ and T(−413)/(GT)_40_ were the most common combinations with frequencies of 22.4%, 30.4% and 19.1%, respectively. It seems that the longer repeats are more often present with a T(−413) allele than the shorter repeats (p < 0.001), however, no linkage disequilibrium (LD) was found (data not shown). In Table 5, the combinations of the (GT)_n_ repeat genotypes and the T(−413)A genotypes are shown. The most prevalent combinations were ML/TA (16.7%), LL/TT (14.9%), SL/TT (12.1%), ML/TT (10.6%) and SM/TA (10.0%), whereas the combination SS/AA was not found. A hypothesis based on earlier human studies was that the combination LL/TT should confer protection against severe malaria. However, analysis with the logistic regression model showed that no combination were more likely to develop severe malaria compared to LL/TT (p > 0.05).

**Table 5:** **Combinations of the (GT)n repeat and A(−413)T genotypes**

**(GT)n**	**T(−413)A**	**N (%)**	**OR (95% CI)**	**P value**
SS	AA	0 (0.0)	-	-
SS	AT	3 (0.6)	1.77 (0.15-21.34)	0.65
SS	TT	23 (4.3)	0.63 (0.22-1.84)	0.40
SM	AA	3 (0.6)	<0.001 (<0.001- > 999.99)	1.0
SM	AT	54 (10.0)	1.01 (0.46-2.22)	1.0
SM	TT	22 (4.1)	1.12 (0.42-3.37)	0.75
SL	AA	2 (0.4)	<0.001 (<0.001- > 999.99)	1.0
SL	AT	21 (3.9)	0.39 (0.09-1.58)	0.19
SL	TT	65 (12.1)	0.70 (0.33-1.50)	0.36
MM	AA	37 (6.9)	0.66 (0.27-1.58)	0.35
MM	AT	27 (5.0)	0.70 (0.24-2.04)	0.52
MM	TT	18 (3.4)	0.59 (0.14-2.45)	0.47
ML	AA	9 (1.7)	1.43 (0.28-7.21)	0.66
ML	AT	90 (16.7)	1.30 (0.66-2.57)	0.45
ML	TT	57 (10.6)	0.71 (0.31-1.62)	0.42
LL	AA	2 (0.4)	<0.001 (<0.001- > 999.99)	0.99
LL	AT	25 (4.7)	0.13 (0.02-1.08)	0.06
LL	TT	80 (14.9)	Reference	-

Frequencies of the T(−413)A genotypes combined with the genotypes of the (GT)_n_ repeats. The combinations ML/AT, LL/TT, SL/TT, ML/TT, SM/AT were the most prevalent. No significant association with severity of malaria was found by analysis with logistic regression models, adjusted for age and sex. Significance level p < 0.05.

## Discussion

This study investigated the possible association between *HMOX1* polymorphisms and severity of malaria. Based on earlier studies, it was hypothesized that certain polymorphisms in the promoter of the *HMOX1* gene encoding HO-1 could confer protection against severe malaria. However, this study could not provide evidence for such an association.

The (GT)_n_ repeat length polymorphism have been studied extensively in the past decade, relating short or long repeat alleles to the risk of many different diseases [38,39]. In this current study, the alleles ranged from 21 to 46 repeats, which is similar to findings in studies in Angola and The Gambia [32,34]. However, there were exceptions; short alleles down to 13 repeats were found in The Gambia [34] and repeats > 41 found in the present study were not found in Angola [32]. The majority of the alleles had lengths of 26, 29/30 and 39/40 repeats, consistent with the study in The Gambia [34]. In Angola, the distribution was slightly different, with the most frequent alleles being 23, 29 and 38 repeats. The differences in the alleles around 29/30 and 38–40 repeats found in the present study as compared to the two previous studies, could be due to small discrepancies in the analysis of the (GT)_n_ nucleotide repeats. However, this did not affect the length-category analysis since both the 29 and 30 repeat alleles were categorized as medium-sized and 38–40 as large. Studies outside Africa have found much lower frequencies of the 39-repeat allele than the current study of less than 3% in Japan [28], Thailand [35] and Greece [31], and absent in a study in Myanmar [33]). In contrast, the allele with 23 repeats was more prevalent in the studies outside Africa with frequencies up to 30% [28,31,33,35] compared to the 6% found here. In a Brazilian study, the frequencies of both the 23- and 39-repeat alleles were low (<2%), whereas alleles with 28–30 repeats all had high frequencies (>65%) [44].

In both the Myanmar study and the two African studies in Angola and The Gambia, short repeat alleles were positively correlated with severity of malaria. Studies in Thailand and Brazil have shown conflicting results [35,44]. In the Brazilian study, long (GT)_n_ repeats were associated with symptomatic malaria, however, the patients were mainly infected with *P. vivax* and only five severe cases were included [44]. In Thailand, no association between the (GT)_n_ repeats and severity of malaria was found, however, limited sample size in some groups might have influenced results and furthermore, the study group consisted of both *P. falciparum* and *P. vivax* infected patients [35]. Although an association between the (GT)_n_ repeat polymorphism and severity of malaria has been shown in two other African populations, this study could not confirm such association. In The Gambia, the allelic and genotypic distributions were different from this current study Thus, the severe malaria patients with short repeat alleles were more prevalent in The Gambia than in Ghana (50% vs 19%), and the long repeat alleles more prevalent in Ghana (43% vs 26%) [34]. This was also reflected in the genotypes, with frequencies of 28% (S/S) and 8% (L/L) in the severe malaria patients in The Gambia compared to frequencies of 5% and 20% in Ghana, respectively. The patient sample size was equivalent to the Gambian and Angolan studies; however, differences in study population, study design, age, or malaria transmission might influence results.

As with pro-inflammatory cytokines for example, excessive levels are cytotoxic [34] and the optimal levels of HO-1 might be a balancing act since both low and very high levels of the enzyme are associated with cytotoxic effects [21,22,52,53]. *HMOX1* has been associated with several diseases [38,39], which may have blurred a possible selective force of malaria on *HMOX1*.

Two single nucleotide polymorphisms (SNPs) in *HMOX1* were also determined. No analysis was done regarding the G(−1135)A SNP, since none of the groups were in Hardy-Weinberg equilibrium. The T(−413)A SNP has been associated with differences in promoter activity, however, it has not been studied as extensively as the (GT)_n_ repeat length polymorphism. The frequency distribution of the T(−413)A SNP reported here is similar to previous findings in a Chinese population [30] and a study based on North Americans and Europeans [54]. However, it differed significantly from a Japanese population where the AA genotype was more prevalent than in the Ghanaian population (26.9 vs 9.7%) whereas the TT genotype was more prevalent than in the Japanese population (49.3% vs 24.5%) [27]. In contrary to our initial hypothesis, no association between genotypes and malaria severity was found.

When alleles of the T(−413)A SNP and (GT)_n_ repeat alleles were considered together, long repeats were mostly found with the T(−413) allele, which is consistent with the findings of the Japanese study [27]. However, there was no linkage disequilibrium between the alleles. Furthermore, the T(−413)A and (GT)_n_ repeat category genotypes were combined; no association with severity of malaria was found. Thus in summary, based on the findings of this study together with the fact that previously found associations between malaria and *HMOX1* have shown effects in opposing directions suggest that malaria does not seem to be a major selective force on the polymorphisms of the *HMOX1* promoter.

## Conclusion

The GT)_n_ repeat allele frequencies found in this study are similar to those of other African studies. However, the association between the (GT)_n_ repeat alleles and severity of malaria was not confirmed in this well-characterized Ghanaian population [45]. Furthermore, the A(−413)T SNP showed no association with severity of malaria alone or in combination with the (GT)_n_ repeat alleles. In this population, the polymorphisms of the *HMOX1* promoter are not associated with severity of malaria, and another selective force may be influencing these alleles.

## Abbreviations

CO: Carbon Monoxide
HO: Haem Oxygenase
HMOX1: Haem Oxygenase 1
LD: Linkage Disequilibrium
L: Long
MHD: Microsomal Haem Degradation
M: Medium
PCR: Polymerase Chain Reaction
RFLP: Restriction Fragment Length Polymorphism
S: Short
SNP: Single Nucleotide Polymorphism
